# On Dislocations, Simple and Compound, of the Ancle-Joint

**Published:** 1820-04-01

**Authors:** 


### *On Dislocations, simple and compound, of the Ancle-Joint.* By Astley Cooper, Esq. F. R. S.

[ Surgical Essays, Part II. ]

There are few accidents incidental to the lower extremity, which create so much anxiety on the part of the practitioner, or are so pregnant with danger of "life or limb" to the patient, as compound dislocations of the ancle-joint. Mr. Cooper has therefore done the surgical department of the profession a great service, by embodying the results of his own extensive experience, and collecting, from various quarters, the experience of others, on this interesting point of Surgery. We shall endeavour to lay before our brethren a comprehensive, yet concise analysis of this part of Mr.

Cooper's work, of which the subject in question forms the prominent and most important article.

Every surgeon knows that Nature has strongly protected the ancle-joint from dislocations, by the deep socket formed by the bones of the leg into which the ball of the astragalus is received, and by strong ligaments passing from the tibia and fibula to the bones of the tarsus. The fibula, in particular, is so firmly bound to the os calcis and astragalus by three excessively strong ligaments, that it is more frequently fractured than dislocated. Mr. Cooper has known the tibia thrown out in three different directions — forwards, inwards, and outwards.

1. The most frequent is *inwards*; in which case, the tibia has its internal malleolus so forcibly projecting against the integuments as to threaten their bursting. The foot is thrown outwards, its inner edge resting upon the ground, with a deep depression about three inches above the outer ancle, while a general tumefaction, from extravasation, surrounds the joint. We shall pass over the internal appearances on dissection, in order to introduce Mr. Cooper's mode of reduction.

"For the reduction of this dislocation, the patient is to be placed upon a mattress properly prepared, and is to rest on the side on which the injury has been sustained; he is then to bend the leg at right angles with the thigh, so as to relax the gastrocnemii muscles as much as is possible, and an assistant grasps the foot, and gradually draws it into a line with the leg. The surgeon fixes the thigh and presses the tibia downwards, and thus forces it upon the articulating surface of the astragalus. Great force is required if the limb be placed in the extended position, from the resistance the gastrocnemii give; and it is pleasing to observe, after most violent attempts, a well-informed surgeon gently bend the limb, and, under a comparatively slight extension, return the parts to their natural situation." p. 98.

After reduction, the limb should be kept on its outer side in the bent position, with the foot well supported; while a many-tailed bandage, wetted with an evaporating lotion, is placed over the part, to prevent its slipping from its place. Two splints are then to be applied, the outer one, on which the limb rests, having a foot-piece to give support to the foot. General or topical bleeding, with open bowels, according to the degree of consequent inflammation, will, of course, be employed. From ten to twelve weeks will elapse before the patient has the free motion of his foot, and much friction and passive movements are necessary.

II. *Simple Dislocation of the Tibia forwards.* The foot appears much shortened; the heel lengthened; the toes pointed inwards. Dissection shews the tibia resting on the upper surface of the os naviculare, and os cuneiforme internum, quitting the articulatory surface of the astragalus. The fibula, as in the former case, is broken; and in this instance, its fractured end advances with the tibia. This dislocation arises in consequence of the body falling backwards, whilst the foot is confined; or, from jumping from a carriage in rapid motion, with the toe pointed forwards.

*Reduction.* The patient is to be placed in bed on his back. One assistant grasps the thigh at its lower part, and draws it towards the body, and another pulls the foot in a line a little before the axis of the leg, and the surgeon pushes down the tibia to bring it into its place. A many-tailed bandage must be lightly applied, wet with evaporating fluids. The local and constitutional treatment, the same as in the former case. The best position is the heel resting on a pillow in bed, with a splint properly guarded on each side of the leg, with a foot-piece to keep the foot well supported at right angles with the leg. In five weeks, the fibula will be united, and the patient may then be taken out of bed.

The tibia may be partially luxated forwards, so as to rest half on the os naviculare, and half on the astragalus. The fibula is broken; the foot appears but a little shortened; nor is there any considerable projection of the heel. The foot is pointed downwards, and a difficulty is experienced in putting it flat to the ground. The heel is drawn up, and the foot is nearly immoveable. In these cases, however slight the accident may appear at first, there is the utmost necessity that the foot should be returned into its natural position; for if neglected in the beginning, severe inflammation and tension will prevent even a forcible extension being afterwards useful. If the reduction be still longer neglected, the changes in the state of the muscles, and the union of the fibula will render all subsequent attempts at replacement unavailing. The same steps are here to be taken as in complete luxation forwards, already described.

III. *Simple Dislocation of the Tibia Outwards.*—This is the most formidable of all, being attended with more contusion of the integuments, laceration of the ligaments, and injury of the bone. The foot is thrown inwards, its outer edge resting on the ground, the malleolus externus projecting through the integuments, and forming so decided a prominence, that the true nature of the injury can hardly be mistaken. In this accident, the malleolus internus is obliquely fractured, and separated from the shaft of the bone, the fracture sometimes extending obliquely through the articular surface of the tibia, which is thrown forwards and outwards upon the astragalus. The latter is occasionally fractured, and the lower extremity of the fibula broken into several splinters. The deltoid ligament escapes, but the capsular ligament is torn on its outer part. This accident happens either by the wheel of a carriage passing over the leg, or by the foot being twisted inwards, in jumping or falling.

"The mode of reduction consists, in placing the patient upon his back, in bending the thigh at right angles with the body, and the leg at right angles with the thigh; the thigh is then grasped under the ham by one assistant, and the foot by another; and thus an extension is made in the axis of the leg, whilst the surgeon presses the bone inwards towards the astragalus. The limb, in the simple dislocation, is to be laid upon its outer side, resting upon a splint, with a foot-piece, and a pad is to be placed upon the fibula, just above the outer angle, and extending a few inches upwards, so as in some measure to raise that portion of the leg, and support it so as to prevent the tibia and fibula slipping from the astragalus, as well as to lessen the pressure of the malleolus externus upon the integuments, where they have sustained injury." 103.

The local and general treatment will be the same as in the former case, except that more active depletion will be necessary to meet the greater inflammation which succeeds.

### COMPOUND DISLOCATIONS OF THE ANCLE-JOINT.

The only difference between these and simple dislocations, already described, is, that the joint is laid open by a wound in the integuments and ligaments, opposite to the laceration of the skin, by which the synovia escapes, and the bones protrude. But the bones being replaced, and the same means employed, as in simple dislocations, what results? The synovia escapes through the wound, and in a few hours, inflammation is kindled. As the inflamed and irritated vessels of the part become now more turgid with blood, the synovial secretion and discharge are increased; the ligaments, and even the articular bones inflame; the suppuration, in five or six days, becomes established in the synovial membrane, and ultimately in the ligaments and periosteum, when an abundance of matter flows from the wound.

Under this process the cartilages become partially or wholly absorbed, in a slow manner, and attended with severe constitutional irritation, often ending in exfoliation of the extremities of the bones. When they are completely absorbed, granulations spring from the surface of the bones, and from the synovial membrane, to inosculate and fill up the interspace. This inosculation of granulations, and the process of adhesion between the bones, need not lead to permanent anchylosis, if passive motion be begun as soon as cessation of pain and inflammation will permit. Motion, indeed, will not be restored entirely; but, in general, with very little diminution.

It is natural to expect, that such a local injury would induce considerable constitutional irritation and fever; and such is the fact. In two or three days after the accident, sometimes sooner, there will be pain in the head and back; furred tongue, white, brown, or nearly black, according to the degree of fever; disordered stomach; inappetency; cessation of secretion in the mucous surfaces of the intestines, and in the liver, with its concomitant symptom, costiveness; hot, dry, and constricted skin; high-coloured and scanty urine; increased vascular action; derangement of the nervous system, &c.

"The first question which arises upon this subject is the following: *Is amputation generally necessary in compound dislocations of the ancle?* My answer is, certainly not. Thirty years ago, it was the usual practice to amputate limbs for this accident, and it was then thought absolutely necessary for the preservation of life by some of our best surgeons; but so many limbs have been of late years saved, indeed, I may say, so great a majority of cases, that such advice would now be considered not only injudicious, but cruel. It is far from being my intention to state that amputation is never required; but only to observe, that in by far the greater number of these accidents, this operation is unnecessary." 107.

Before exhibiting proofs of this proposition, our author lays down the mode of treatment to be pursued in these cases.

When the surgeon is called to an accident of this kind, the arrest of hæmorrhage, if there be any large artery divided, (which is rarely the case) forms the first indication. The posterior tibial artery, our author never saw wounded in these dislocations. If the anterior tibial be torn, it is to be secured at once. The protruded extremity of the bone is next to be most carefully washed, as the least quantity of extraneous matter, returned into the joint, would aggravate the inflammation. If the bone be shattered, the finger is to be passed into the joint, and the detached pieces removed, in the gentlest manner possible. If the wound will not admit the finger easily, it should be enlarged *upwards* with the scalpel.

"The integuments are sometimes nipped into the joint by the projecting bone, and it cannot be reduced when this is the case, without making an incision upwards, to allow of the skin being brought from under the bone; and when the edges of the incised wound are afterwards brought together, no additional evil arises from the extension of the wound." 109.

The mode of reducing the bone is, in all other respects, precisely similar to that employed in simple dislocations, according as the luxation is inwards, forwards, or outwards. When the bone has been reduced, a piece of lint is to be dipped in the blood, and applied wet over the wound, where it forms a coagulum, and, as far as Mr. Cooper has seen, the best covering. A many-tailed bandage is then to be applied, and to be kept constantly wetted with spirits of wine and water. A hollow splint, with a foot-piece at right angles, is to be applied on the outer side of the leg, when the dislocation is inwards, and the limb is to rest upon its outer side. Where the dislocation is outwards, the best position is on the heel, with a splint on each side, an aperture in the splint being made opposite to the wound. The gastrocnemius muscle is, of course, to be relaxed by a slight bend of the knee-joint, and the foot carefully prevented from pointing, and kept steadily at right angles with the leg, otherwise the limb will be useless when the wound is healed. The patient is to be placed on a mattress, and a pillow is to reach from half way above the knee to beyond the foot, while another is to be rolled under the hip, to support the upper part of the thigh bone.

As the constitution has ultimately to undergo a great trial of its powers, blood-letting and purging, Mr. Cooper states, must be carefully managed, and that which is judged necessary, in this way, should be done at the very first hours* of the accident, before the adhesive inflammation arises.

"There cannot be a worse practice, when a limb has been placed

* Mr. Cooper says the "first *hour* of the accident;" but we cannot help thinking that this is an incautious expression. Those who have been in the habit of seeing these serious accidents of joints, or other parts, on the spot, know that there is very generally a recoil, rather than a deploy of the vital powers, during the first *hour* subsequent to a violent injury, often, indeed, amounting to faintness, paleness, and vomiting. Surely this would not be the period for general blood-letting. We have therefore modified Mr. Cooper's expression.

in a good position, and adhesion is proceeding, than to disturb the processes of Nature by the frequent changes of position which purges produce; and I am quite sure that I have seen patients destroyed by their frequent administration." 110.

We have, indeed, seen some melancholy effects result from motion in compound, and even in simple fractures, where that motion was inevitable, as in the case of men wounded in battle; and often have we had occasion to admire the wonderful powers of Nature in repairing injuries, under circumstances of disaster, of which the civil surgeon can form but an inadequate idea! A tempest succeeding a naval engagement, and a precipitate retreat after a conflict in the field, present instructive lessons to the eye of the medical officer, respecting the point in question. We shall quote the following passage relative to the "secondary treatment," and then make a remark or two of our own.

"If the patient complains of considerable pain in the part in four or five days, the bandage may be raised to examine the wound; and if there be much inflammation, a corner of the lint should be lifted from the wound, to give vent to any matter which may have formed; but this ought to be done with great circumspection, as it is in danger of disturbing the adhesive process, if that be proceeding without suppuration. By this local treatment, it will every now and then happen that the wound will be closed by adhesion, but if in a few days it be not, and suppuration take place, the matter should have an opportunity of escaping; and the lint being removed, simple dressings should be applied. After a week or ten days, if there be suppuration, with much surrounding inflammation, poultices should be applied upon the wound, leeches in its neighbourhood, and upon the limb, at a distance, the evaporating lotion should still be employed; but as soon as the inflammation is lessened, the poultices should be discontinued, as they encourage too much secretion, and relax the blood-vessels of the part, so as to prevent the restorative process." P. 111.

We have had some experience in the accident under consideration, and in a class of still more formidable injuries of the joints—gun-shot wounds. It is with reluctance that we differ in opinion from a man of Mr. Cooper's acknowledged talents and experience; but absolute perfection is not the lot of even the most gifted mortals. While then we agree with our author, as to the caution against carrying general blood-letting too far, in injuries of this kind, we cannot but think that his omission of *early local* bleeding is a defect in his plan of treatment. We are convinced, from observation, that the adhesive process, which he desires, is promoted, and that the suppuration, which he dreads, is checked by vigorous local bleeding—a species of depletion, too, which does not subject the constitution to the hazard of a depression that may render it unequal to the ultimate trial which it is to undergo. We could wish that Mr. Cooper would consider the sentiments of Dr. Hennen on this subject, and take, for example, the case of Colonel R——,* who was shot through the knee-joint at the battle of Waterloo. This officer, besides one general bleeding, had upwards of two hundred leeches to the neighbourhood of the joint, in the first seven days of treatment, while scarcely any food was allowed.

"On the evening of the seventh day (says Dr. Hennen) some slight rigors, and the appearance of the knee, indicated the formation of matter, which occasioned the change of application. *The very rigorous treatment employed during the inflammatory stage, limited this formation considerably.* During the whole period from the infliction of the wound to the change of external applications, on the seventh day, which includes the period of inflammation, the quantity of blood lost, including the general bleeding, which preceded the application of the leeches, amounted, by calculation, to 235 ounces. The quantity of food taken during these seven days, was so small, that, in comparison, Valsalva's diet was excess; but to this certainly much of the preservation of the limb was due."

The medical officers, in fact, of our fleets and armies, have long been taught by extensive observation, that the most effectual method of enabling the constitution to bear up against serious injuries of joints or other parts, is to keep down, by all possible means, the *local* irritation and inflammation; and that indication is best fulfilled by repeated applications of leeches, in great numbers; by cold applications; by quietude; and by starvation. If we stand by, and suffer the local inflammation to kindle up general fever and irritation, and consequently to produce extensive suppuration, it is we ourselves who then subject the constitution to the severe trial which we ought to have endeavoured to ward off, by crushing the cause at its source. These are not theoretical speculations, but deductions from facts. The principle which we are remonstrating against, is well known to have been long applied to fevers themselves. We were gravely cautioned by the most experienced practitioners to avoid depletion at the beginning of fevers, in order that the constitution might be able to go through the long state of exhaustion which was to succeed the period of excitement. It is hardly necessary to state, that it is now abundantly proved, that the most effectual

* Page 162 of Dr. Hennen's work on Military Surgery.

mean of obviating the said exhaustion, is *early* depletion, so as to moderate the excitement. The very same principle is applicable to violent local injuries—the more the local irritation and inflammation are kept in check, by local and general depletion, &c. the greater the chance of adhesive reunion, and the less the extent of local suppuration and general fever.

But we must now resume the thread of our analysis. If the cure proceeds favourably, the wound is healed with little suppuration. "If less favourably, a copious suppuration takes place, the wound is longer in healing, and exfoliation of the extremity of the bone still further retards the cure." If this be not an unequivocal indication for early local bleeding, we know not what is; and we are surprised, that the superior penetration and discernment of Mr. Cooper should have so much overlooked what we consider the paramount measure.

The motion of the joint is not always lost; but sometimes, in a great degree, restored. "This depends upon the greater or less extent of suppuration or ulceration." Under the most favourable circumstances, the patient may be about, on his crutches, in three months; but, at other times, the period of recovery is much longer.

Mr. Cooper appears to have taken much pains in procuring information on the subject under review, from various surgical friends, and introduces communications from Mr. (now Dr.) Lynn of Woodbridge, Mr. Battley of London, Mr. Richards of Seal, Mr. Rowley, Dresser at St. Thomas's Hospital, Mr. Clarke of Lincolns-Inn Fields, Mr. Somerville of Stafford, Mr. Scarr of Bishop's Stortford, Mr. Abbot of Needham Market, Mr. Ransome of Manchester, Mr. Chandler of Canterbury, Mr. Hammick of Plymouth Hospital, Mr. Wickham of Winchester, Mr. Smith of Bristol, Mr. Averill, Dresser at Guy's Hospital, Dr. Kerr of Northampton, Dr. Rumsey of Amersham, Mr. Hicks of Baldock, Mr. Cooper of Brentford, Mr. Carden of Worcester, Mr. Fletcher, Dr. James Lynn; and then introduces several cases of his own. Finally, he winds up this long and interesting paper with observations on those cases which *do* require amputation. Our limits will not permit us to notice all the communications, nor all Mr. Cooper's own cases:—those which are passed over, are more so from necessity on our parts, than from any want of interest on their's.

*Case.* Mr. Battley relates the case of an insane gentleman, who jumped from a two pair of stairs window into the street; received a compound dislocation of the ancle-joint, with shattered astragalus; yet walked up stairs in that state! The unconnected portions of bone were removed; the tibia replaced; the dressings already described applied, with cold lotions. On the third or fourth day, "considerable inflammation appeared in the joint, and greatly increased the previous irritable state of his constitution."

In this case, *leeches* and general blood-letting were employed, at the period above mentioned; extensive suppuration ensued, and continued for nearly two months, when it began to decline, and healthy granulations to sprout up. In nine months he was out of the sick list, with anchylosed joint.

*Case.* Mr. Clarke and Mr. Cooper attended a gentleman in October 1817, with compound dislocation of the ancle-joint, and fracture of the tibia. The end of the bone projected two or three inches, and was tightly embraced by the skin. To effect reduction, an incision was made in the integuments; the usual dressings were applied; bleeding was had recourse to, with purgatives and saline medicines.

"Symptoms of great constitutional excitement naturally arose from so severe a local injury. Abscesses formed on the leg, and some exfoliations materially retarded the cicatrization of the wound, and produced considerable exhaustion of his strength."

Recovery took place.

An interesting case is related by Mr. Scarr, of a very severe compound dislocation, successfully treated; but we dare not dwell on the particulars.

*Case.* A patient of Mr. Abbot's, nearly 70 years of age, corpulent, intemperate, and gouty, was thrown violently to the ground, and suffered a compound dislocation of the tibia, the end of which was forced through the integuments nearly four inches, the wound being large and circular. In the struggle to stand erect, he rested his weight on the end of the bone, "which was covered with sand and dirt; the cavity of the articulating surface of the joint was filled with blood and sand, the fibula fractured a few inches above the joint, and the foot completely turned outwards." In such a situation this wretched inebriate was conveyed several miles in an open cart, during a cold night, and continued seven hours without surgical assistance! Mr. Jefferson, now of Islington, was with Mr. Abbott, and amputation was proposed, but obstinately rejected by the patient. The surfaces were carefully, and expeditiously as possible, made clean with warm water, and the reduction was easily accomplished. One general bleeding was practised, with laxatives and milk broth only for food. Next day, as some inflammation appeared rising, *leeches* were applied, " a small distance from the inflamed part; they bled freely, and afforded ease." This case had a most rapid recovery, and without the least sinister accident. Mr. Abbot has, since the foregoing accident, treated a variety of compound fractures in the same way, and with similar success.

We cannot but attribute a share, at least, of the success in this old man's case, to the local bleeding, the good effects of which are recorded in Mr. Abbot's words. Indeed, we are free to confess, that Mr. Abbot's plan of treatment is much more to our minds than any set forth in this volume, not even excepting Mr. Cooper's own cases.

Mr. Cooper remarks, that cases occasionally occur, where it may be judicious practice to saw off the end of the projecting tibia, especially where there is much difficulty experienced in reducing it—where it is broken obliquely, so that when reduced, it will not remain upon the astragalus —when the spasmodic contractions of the muscles are violent—and when the bone is much shattered. The following cases are illustrative of this point.

*Case.* A boy, aged 13 years, was carried to Guy's Hospital to have the leg amputated, in consequence of a severe injury from a boat having fallen on it. Mr. Cooper found the case as follows:

" A large wound was seen at the outer ancle, through which the tibia and a fractured extremity of the fibula projected; one inch of the malleolus externus remained attached to the astragalus by its natural ligaments; the foot was turned inwards, so as to be able to be made to touch the inner side of the leg; and from the muscles being no longer on the stretch, the foot was very loose and pendulous. I tried to reduce the limb, but found that the bone could only by great violence be brought on the astragalus, and that it immediately slipped from its place. The case was, therefore, as regarded the state of the parts, the most unfavourable possible; and those around me urged an immediate amputation; but seeing the youth, and the character of health which the boy bore, I thought I should not be justified in dooming him probably to a life of mendicity, and I determined to try to preserve the limb. Finding that the lower end of the fibula, although still connected by ligament, was very loose and moveable, I removed it with the scalpel; I then sawed off half an inch of the lower extremity of the tibia. When these operations had been accomplished with the greatest care, I reduced the bones, and they maintained their situation, as there was no force of muscular action upon them, on account of the shortening of the bones. Lint, wetted with the person's blood, was then applied, with adhesive plaisters over it, and the leg was put in splints and placed on the heel. Scarcely any constitutional irritation occurred; the wound and ancle-joint secreted but little matter, and gradually healed." P. 142.

Among the list of contributors to Mr. Cooper's work, we find the venerable patriarch of medico-chirurgical practitioners, Dr. Kerr, of Northampton, "who, (to use our author's expressions) at the age of more than *eighty*, still continues to practise his profession with all the ardour of youth, and with a strength of intellect which has been seldom surpassed."

Dr. Kerr. who must have seen many revolutions in surgery as well as in politics, during a reign of nearly sixty years, asserts that "it has *uniformly* been his practice to take off the lower extremity of the tibia, and to lay the limb in a state of semiflexion upon splints, &c." Our readers are aware that Mr. Cooper states the general practice, 30 years ago, to be amputation of the limb, in these cases; and as Dr. Kerr has been in practice more than double that time, we think humanity should have led him, some half a century ago, to remonstrate against a useless dismemberment, and point out the present mode of saving so many limbs.

The case related by Dr. Rumsey, of Amersham, is extremely interesting and well stated; but we have not room for an analysis of it, without spoiling most of its prominent features.

The following case, related by Mr. Hicks, of Baldock, will sufficiently illustrate the plan of removing the protruded portion of tibia.

*Case.* A coachman's ancle was dislocated and the femur fractured by the upsetting of a coach. The foot was turned upon the outside of the leg; the tibia and fibula protruded four inches through the integuments; the head of the fibula was fractured, and several small portions of it remained within the integuments; the end of the tibia had some small portions chipped off it, appearing as if it had been ground by the side of the coach; the foot, in fact, appeared almost separated from the leg, and the ends of the bone were covered with dirt. The ends of the tibia and fibula were sawed off, and then the fractured portions of the latter were searched for with the finger, and extracted by the aid of the bistoury. The dislocation was then reduced, the usual dressings applied, and a proper position enjoined. Antiphlogistic measures were pursued. It is not a little remarkable that "through the whole of the cure the man went on remarkably well, and had little symptomatic fever; pulse constantly below the natural standard, between 60 and 70; skin soft and moist; the action of the intestines was regularly kept up by draughts; the integuments united by the first intention, without the least secretion of pus." In seven weeks the patient was removed to his residence, and required no farther surgical aid. In a few months afterwards the patient paid Mr. Hicks a visit, "walked perfectly well," and the leg was very little shorter than the other.

But, as Mr. Cooper justly observes, cases will occur, in which amputation will be absolutely necessary, "either to preserve the life of the patient, or to prevent his being doomed to the constant necessity of using crutches on account of the deformity and stiffness of the limb." 164. This operation, however, is not always successful; for it is well known that, when amputation was generally practised in compound dislocations of the ancle, "a considerable number died."

Our author thinks that very *old age* is a sufficient reason for amputation; "because the patient is unable to bear the constitutional excitement which the suppurative inflammation of the joint produces." This must be understood as applying to hospital practice in this metropolis, where, to say the truth, operations of all kinds are comparatively unfortunate. Extensive and lacerated wounds may authorize us to remove the limb, as also a very shattered state of the bones. It may be stated generally, that dislocation of the tibia at the outer ancle produces more injury and danger than at the inner, and will more frequently require amputation. When the tibia, in its dislocation outwards, happens to be broken obliquely, and only a small portion of the articulating surface remains on the dislocated extremity of the bone, it will not rest on the tibia, when it is reduced; here amputation is the safest practice. The rupture of either anterior or posterior tibial artery, especially the latter, will be a very unfavourable circumstance, and if coupled with other bad states of the parts, may often prove a very fair apology for amputation.

Mortification of the foot leads, of course, to removal of the limb; generally, however, when the sphacelus ceases to extend. But when mortification results from injury to a blood-vessel, Mr. Cooper thinks that the line of separation is not so necessary a preliminary to the operation as where the limb is dying from a constitutional cause. Our author has seen two successful instances of amputation, during the progress of sphacelus, and the same we have ourselves seen. We have also seen amputation during the ascent of erysipelatous mortification along the thigh terminate safely; but in two instances, apparently similar, the operation failed. It is well known to our surgical readers that our French brethren are bolder, in this respect, than ourselves.

Excessive contusion, as where heavy carriages pass over the joints, and which would lead to extensive sloughing, ought to induce us to operate at once. Even after the attempt to save the limb is made, a profuse suppuration, followed by ulceration of the ligaments, and additional exposure of the joint, will occasionally form a reason for amputation. In the case of tetanus supervening on wounds, our author has seen no good arise from amputation; and we are sorry that our experience, in such instances, is corroborative of Mr. Cooper's observation. Our continental brethren have been more successful, however, and have held out a ray of hope in these desperate circumstances.

The only remaining subject is, dislocation of the tarsal bones, which will not detain us long.

1. *Astragalus.* A simple dislocation of this bone rarely occurs, and a compound one still more seldom. It is a serious accident, being very difficult to reduce; and, in the event of non-reduction, the patient is lame for life. Mr. Cooper lays down no rules for either the simple or compound dislocation.

2. *Bones of the Tarsus.* These are sometimes dislocated from the os calcis and the astragalus.

"There is a transverse joint between these bones, formed by the astragalus and os calcis posteriorly, and by the os naviculare and os cuboides upon the fore part, which support the three ossa cuneiformia. This joint is sometimes, but rarely, dislocated by very heavy weights falling upon the foot, of which the following is an example.

"A man working at the Southwark Bridge had the misfortune to have a stone of great weight glide gradually upon his foot. He was almost immediately brought to Guy's Hospital, and the appearance of the foot was, that the os calcis and astragalus remained in their naturral situations, but the fore part of the foot was turned inwards upon those bones, When examined by the young gentlemen, the appearance was so precisely like that of a *club foot*, that they could not at first believe but it was a natural defect of that kind; but upon the assurances of the man, that his foot, previously to the accident, was not distorted, an extension was made by fixing the leg and the heel; the fore part of the foot was then drawn outwards, and thus the dislocation was reduced. This person was discharged from the hospital in five weeks quite recovered, having the complete use of his foot." 183.

3. *Os cuneiforme internum.* Mr. Cooper has seen two instances of this. In both there was a great projection of the bone inwards, and some degree of elevation, from its being drawn up by the action of the tibialis anticus muscle, and no longer remaining in a direct line with the metatarsal bone of the great toe. In neither case was the bone reduced; but one of the patient's walked with little halting.

The treatment will consist in confining the bone in its place, by first binding it with a roller dipped in spirits of wine and water, with which it must be kept constantly wet.

When the inflammation is subsided, a leather strap is to be buckled around the foot, to keep the bone in its place till the ligament be united with the bone in its natural position.

The minute, and we trust, impartial analysis which we have now laid before the public will enable them to appreciate the merits of Mr. Cooper's work, unbiassed by false colourings of censorious criticism. We are quite confident that every disinterested individual, of correct feeling, will acknowledge the great obligations which the profession owe to Mr. Cooper for thus imparting the fruits of an extensive experience, unwearied industry, and cultivated talent, to his junior and less fortunate brethren. That a few rival candidates for fame and fortune will carp at many parts of these Surgical Essays, is as natural and certain as that sparks should fly upwards. We do not indeed assert that they are entirely unexceptionable; for what work can be so in the infancy of a science, or as the result of one man's experience? We have ourselves pointed out, what we conceive to be, a defect in the treatment of compound dislocations of the ancle joint; but it is in the spirit of candour and independence; and with feelings of the most profound respect and esteem for the ability, zeal, and assiduity of the author.

